# Supplementary material for: Pharmacologic Activation of an Integrated Stress Response Kinase Promotes Mitochondrial Remodeling
Source: bioRxiv. 2023 Mar 12:2023.03.11.532186. Preprint. [Version 1] doi: 10.1101/2023.03.11.532186 (PMC10029010; doi:10.1101/2023.03.11.532186)
Supplement: Supplementary file 1 [file NIHPP2023.03.11.532186v1-supplement-5.pdf]

bioRxiv preprint doi: https://doi.org/10.1101/2023.03.11.532186; this version posted March 12, 2023. The copyright holder for this preprint (which was not certified by peer review) is the author/funder, who has granted bioRxiv a license to display the preprint in perpetuity. It is made available under aCC-BY 4.0 International license.

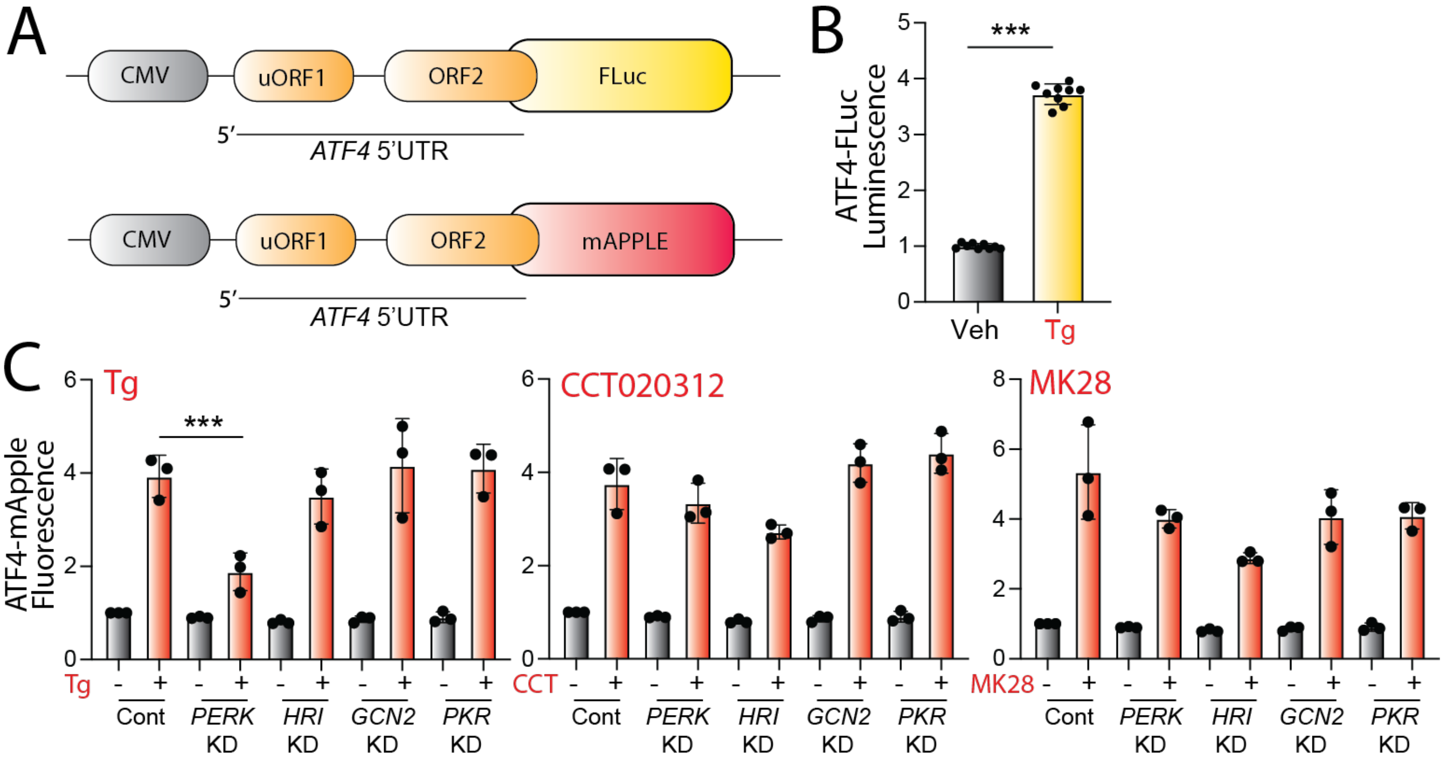

Supplement to Fig. 1. Pharmacologic activation of integrated stress response (ISR) kinases. A. Illustration showing the ATF4-FLuc and ATF4-mApple ISR reporters (Guo *et al.*, 2020; Yang *et al.*, 2022). **B.** Activation of ATF4-FLuc ISR reporter stably expressed in HEK293T cells treated for 3 h with thapsigargin (Tg; 500 nM). Error bars show SEM for n=9 replicates. **C**. Graphs showing activation of the ATF4-mApple ISR reporter stably expressed in HEK293T cells CRISPR-depleted of the indicated ISR kinase and treated for 8 h with BtdCPU (10 μM), halofuginone (100 nM), Erlotinib (25 μM), or Sunitinib (10 μM). Error bars show SEM for n=3 replicates. ***p<0.005 for one-way ANOVA.

bioRxiv preprint doi: https://doi.org/10.1101/2023.03.11.532186; this version posted March 12, 2023. The copyright holder for this preprint (which was not certified by peer review) is the author/funder, who has granted bioRxiv a license to display the preprint in perpetuity. It is made available under aCC-BY 4.0 International license.

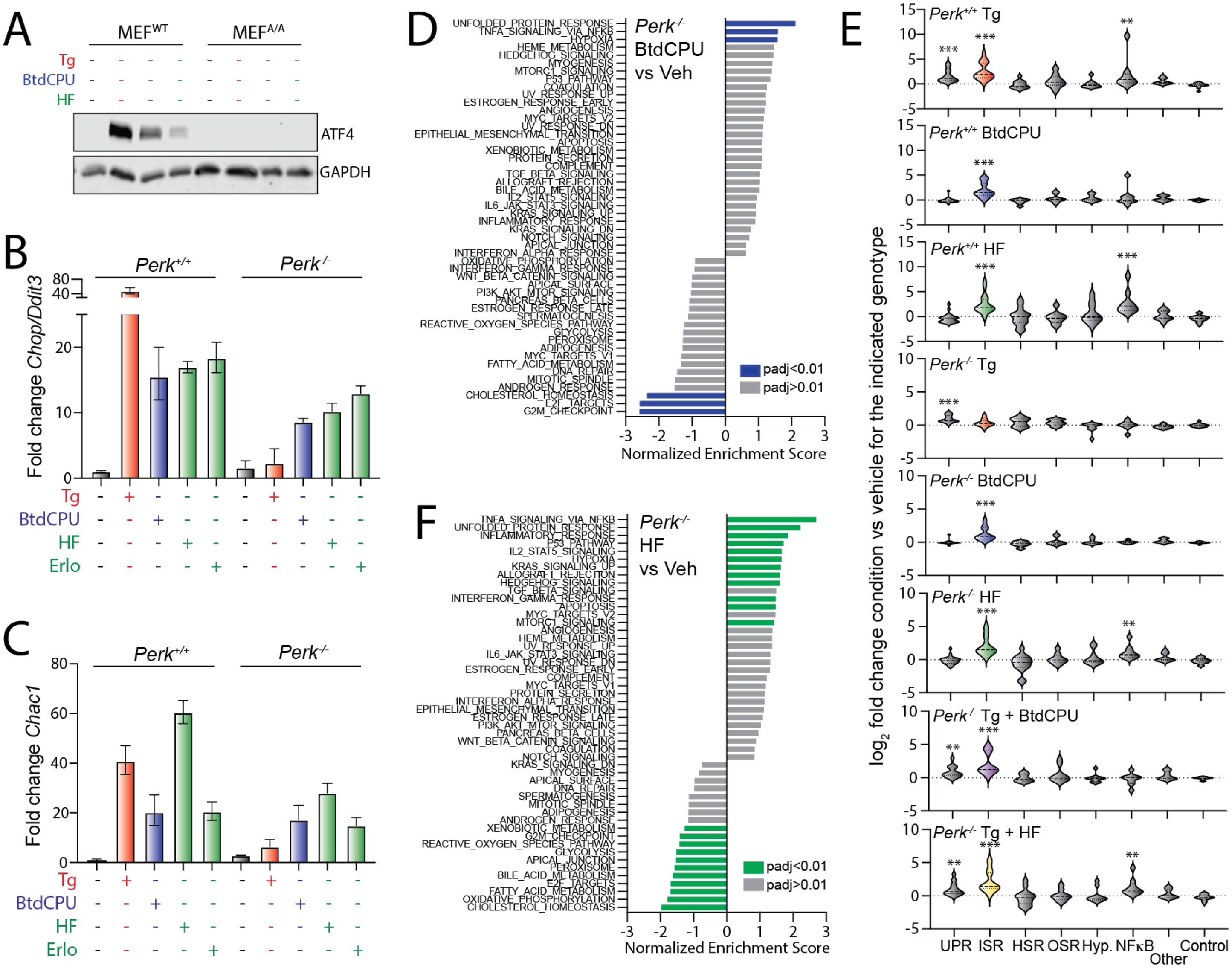

Supplement to Fig. 2. Pharmacologic ISR activators rescue ISR signaling in *Perk*-deficient MEFs. A. Immunoblot of lysates prepared from MEF^{WT} and MEF$^{A/A}$ cells treated for 3 h with thapsigargin (Tg; 500 nM), BtdCPU (10 µM), halofuginone (HF; 100 nM). **B,C.** Expression of the ISR targets *Chop/Ddit3* or *Chac1* in *Perk*$^{+/+}$ and *Perk*$^{-/-}$ MEFs treated for 6 h with thapsigargin (Tg, 500 nM), BtdCPU (10 µM), halofuginone (100 nM), or erlotinib (25 µM), as indicated. Error bars show 95% confidence interval. **D**. Gene set enrichment analysis (GSEA) for hallmark genesets of RNAseq data from *Perk*$^{-/-}$ MEFs treated for 6 h with BtdCPU (10 µM). Full GSEA is included in **Table S3**. **E**. Expression, measured by RNAseq, of genesets comprising target genes of the UPR (IRE1/ATF6), ISR, heat shock response (HSR), oxidative stress response (OSR), hypoxic stress response (Hyp.), NFκB inflammatory response, other stress-responsive genes, and control genes. Geneset are shown in **Table S4**. **F**. Gene set enrichment analysis (GSEA) for hallmark genesets of RNAseq data from *Perk*$^{-/-}$ MEFs treated for 6 h with halofuginone (HF, 100 nM). Full GSEA is included in **Table S3**.

bioRxiv preprint doi: https://doi.org/10.1101/2023.03.11.532186; this version posted March 12, 2023. The copyright holder for this preprint (which was not certified by peer review) is the author/funder, who has granted bioRxiv a license to display the preprint in perpetuity. It is made available under aCC-BY 4.0 International license.

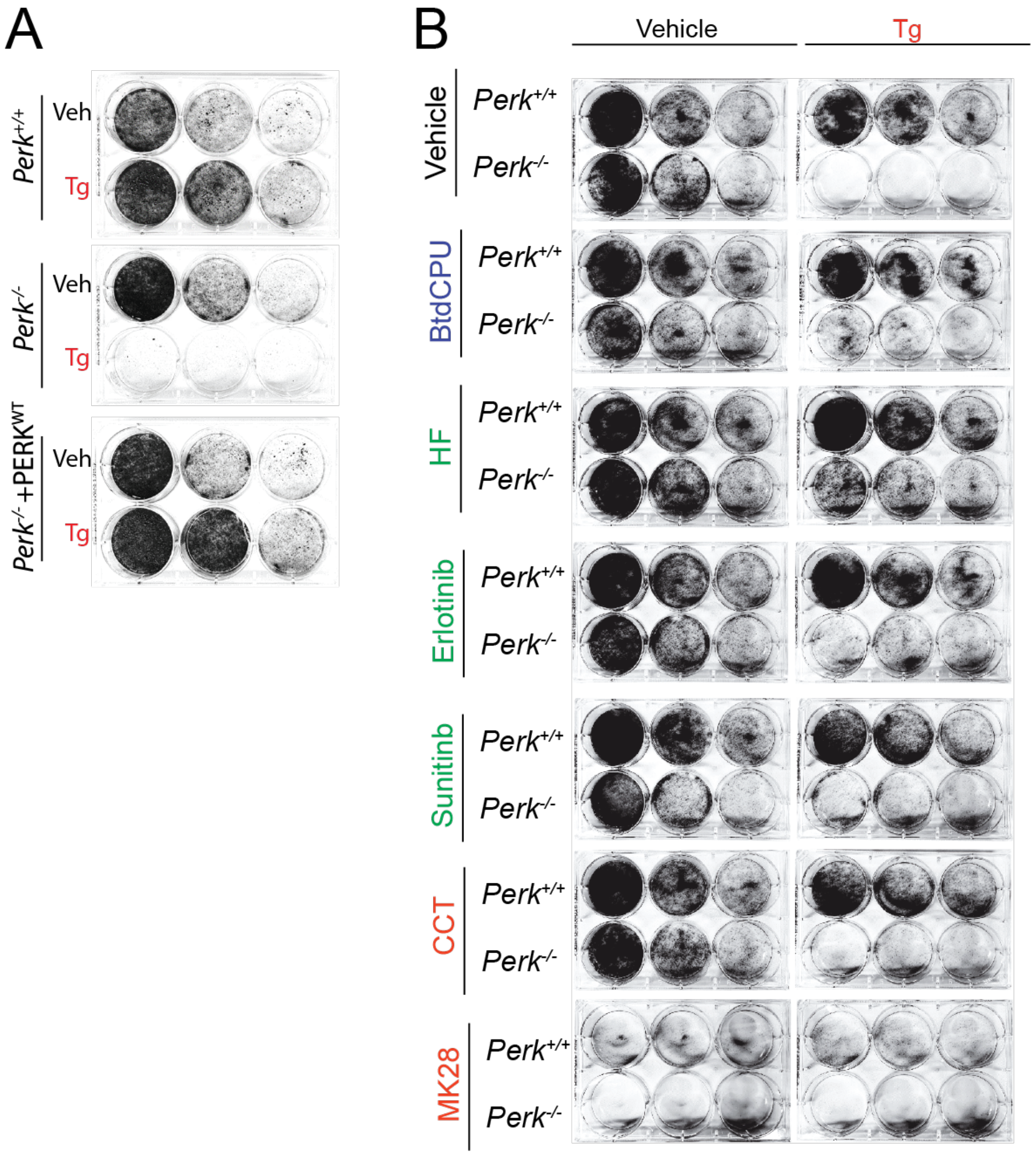

Supplement to Fig. 3. BtdCPU and halofuginone rescue ER stress sensitivity in *Perk*-deficient MEFs. A. Representative crystal violet staining of *Perk*$^{+/+}$, *Perk*$^{-/-}$ and *Perk*$^{-/-}$ MEFs with wild-type *Perk* overexpressed treated for 6 h with thapsigargin (Tg; 500 nM) and then replated and allowed to proliferate in 6 well plates. Crystal violet staining was performed 72 h after replating. **B**. Representative crystal violet staining of *Perk*$^{+/+}$ and *Perk*$^{-/-}$ MEFs treated for 6 h with thapsigargin (Tg; 500 nM) and or BtdCPU (10 μM), halofuginone (100 nM), Erlotinib (25 μM), Sunitinib (10 μM), CCT020312 (10 μM), and MK28 (20 μM). and then replated and allowed to proliferate in 6 well plates. Crystal violet staining was performed 72 h after replating.

bioRxiv preprint doi: https://doi.org/10.1101/2023.03.11.532186; this version posted March 12, 2023. The copyright holder for this preprint (which was not certified by peer review) is the author/funder, who has granted bioRxiv a license to display the preprint in perpetuity. It is made available under aCC-BY 4.0 International license.

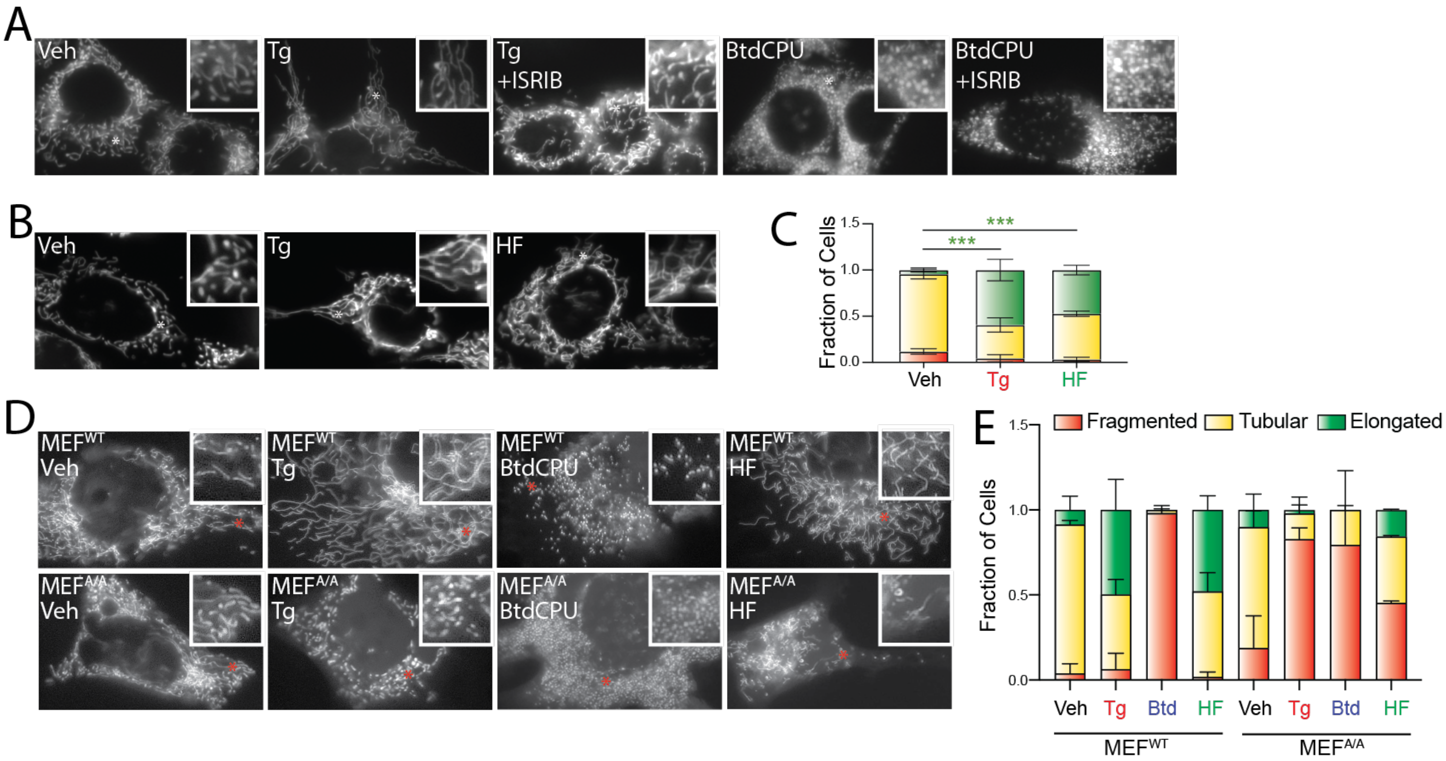

Supplement to Fig. 4. BtdCPU and halofuginone differentially impact mitochondrial morphology. A. Representative images of mitochondrial morphology in MEF cells stably expressing mitochondrial-targeted GFP treated for 3 h with thapsigargin (Tg; 500 nM), BtdCPU (10 µM), and/or ISRIB (200 nM). **B, C**. Representative images and quantification of mitochondrial morphology in MEF cells stably expressing mitochondrial-targeted GFP treated for 3 h with thapsigargin (Tg; 500 nM) or halofuginone (HF; 100 nM). Error bars show SEM for n=2 replicates. ***p<0.005 for two-way ANOVA. (green indicates comparisons between elongated mitochondria fractions). **D, E**. Representative images and quantification of mitochondrial morphology in MEF^{WT} and $MEF^{A/A}$ cells expressing mitochondrial targeted GFP (^{mt}GFP) and treated for 3 h with thapsigargin (Tg; 500 nM), BtdCPU (10 µM), halofuginone (HF; 100 nM). The inset shows 2-fold magnification of the image centered on the white asterisk. Error bars show SEM for n=2 replicates.

bioRxiv preprint doi: https://doi.org/10.1101/2023.03.11.532186; this version posted March 12, 2023. The copyright holder for this preprint
(which was not certified by peer review) is the author/funder, who has granted bioRxiv a license to display the preprint in perpetuity. It is made
available under aCC-BY 4.0 International license.

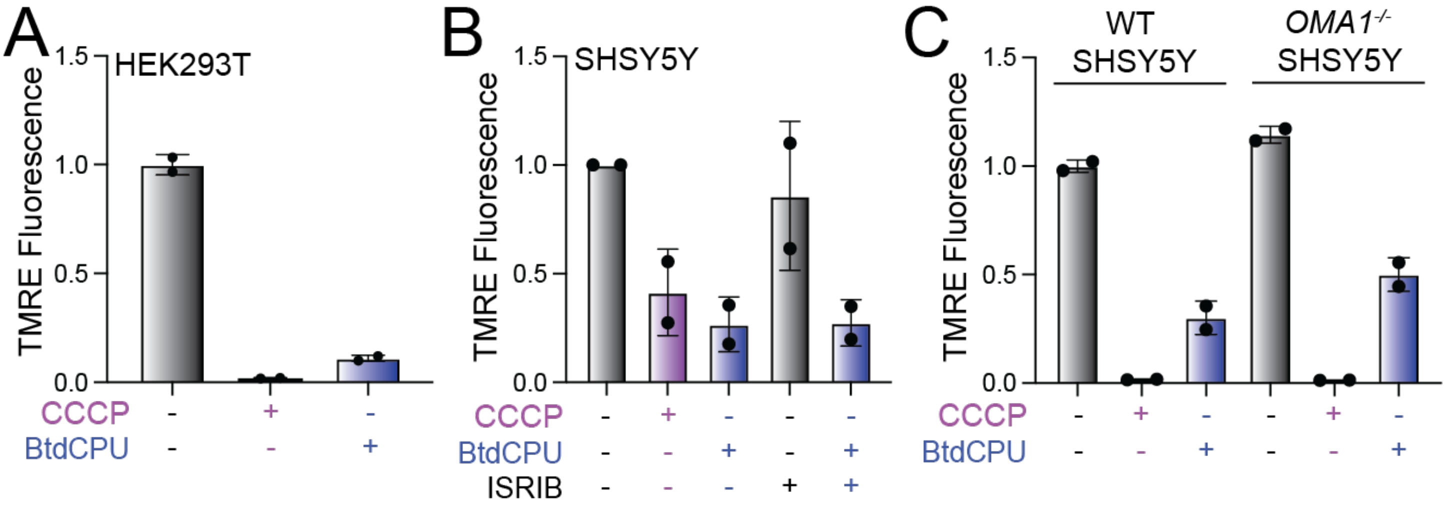

Supplement to Fig. 5. **BtdCPU promotes mitochondrial uncoupling. A**. Mitochondrial membrane potential, measured by TMRE fluorescence, in HEK293T cells pre-treated for 3 h with BtdCPU (10 μM) or for 30 min with CCCP (10 μM). **B**. Mitochondrial membrane potential, measured by TMRE fluorescence, in SHSY5Y cells pre-treated for 3 h with BtdCPU (10 μM) and/or ISRIB (200 nM) or for 30 min with CCCP (10 μM). **C**. Mitochondrial membrane potential, measured by TMRE fluorescence, in SHSY5Y cells or *OMA1* knockout SHSY5Y cells pre-treated for 3 h with BtdCPU (10 μM) or for 30 min with CCCP (10 μM).

bioRxiv preprint doi: https://doi.org/10.1101/2023.03.11.532186; this version posted March 12, 2023. The copyright holder for this preprint (which was not certified by peer review) is the author/funder, who has granted bioRxiv a license to display the preprint in perpetuity. It is made available under aCC-BY 4.0 International license.

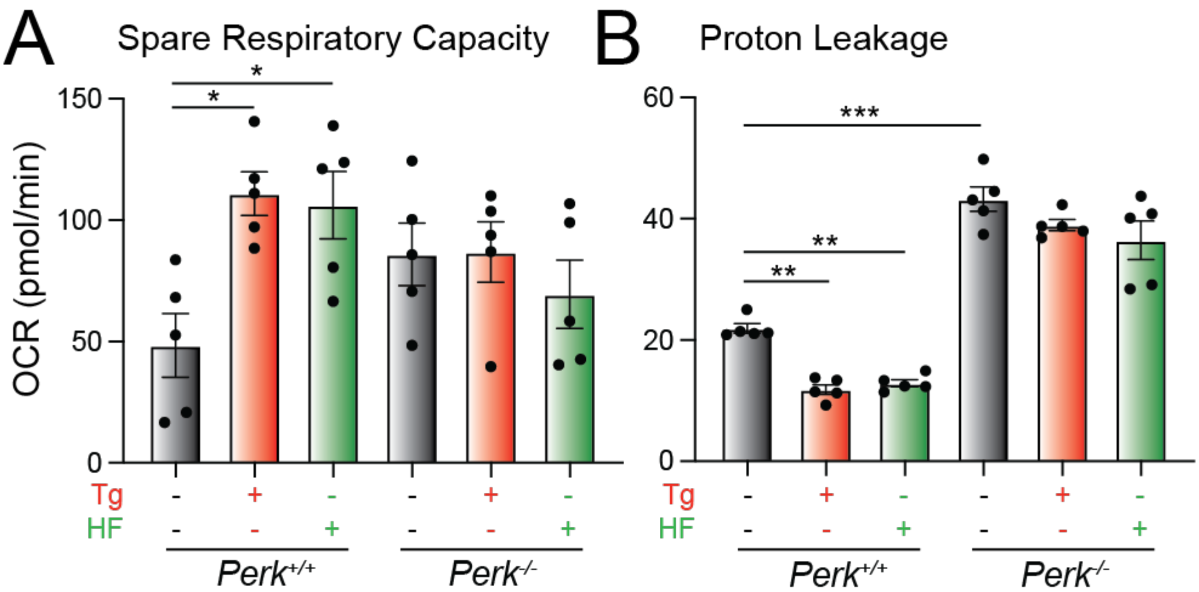

Supplement to Fig. 6. Halofuginone promotes adaptive remodeling of mitochondria respiration in PERK-deficient cells. A,B. Spare respiratory capacity (**A**) and proton leak (**B**) in *Perk*$^{+/+}$ and *Perk*$^{-/-}$ MEFs treated for 3 h with thapsigargin (Tg; 500 nM) or halofuginone (HF, 100 nM) measured from the mitochondrial stress test shown in **Fig. 6C**. Error bars show SEM for n=5 replicates. *p<0.05, **p<0.01, ***p<0.005 from one-way ANOVA.

bioRxiv preprint doi: https://doi.org/10.1101/2023.03.11.532186; this version posted March 12, 2023. The copyright holder for this preprint (which was not certified by peer review) is the author/funder, who has granted bioRxiv a license to display the preprint in perpetuity. It is made available under aCC-BY 4.0 International license.

SUPPLEMENTAL TABLE LEGENDS

Table S1. (Supplement to Figure 2 and Figure S2. Pharmacologic ISR activators rescue ISR signaling in *Perk*-deficient MEFs). DESEQ2 from RNAseq of *Perk*$^{+/+}$ and *Perk*$^{-/-}$ MEFs treated for 6 h with thapsigargin (Tg; 500 nM), BtdCPU (10 μM), and/or halofuginone (HF; 100 nM). The complete RNA-seq data is deposited in gene expression omnibus (GEO) as GSE227134.

Table S2. (Supplement to Figure 2 and Figure S2. Pharmacologic ISR activators rescue ISR signaling in *Perk*-deficient MEFs). Expression, measured by RNAseq, of unfolded protein response (UPR; IRE1/XBP1s and ATF6) target genes and integrated stress response (ISR) target genes, as defined in Grandjean et al (2019) *ACS Chem Biol*, in *Perk*$^{+/+}$ and *Perk*$^{-/-}$ MEFs treated for 6 h with thapsigargin (Tg; 500 nM), BtdCPU (10 μM), and/or halofuginone (HF; 100 nM). The expression of individual genes is shown normalized to that observed in Tg-treated *Perk*$^{+/+}$ MEFs, as described in Grandjean et al (2019) *ACS Chem Biol*.

Table S3. (Supplement to Figure 2 and Figure S2. Pharmacologic ISR activators rescue ISR signaling in *Perk*-deficient MEFs). Geneset Enrichment analysis (GSEA) of RNAseq data from *Perk*$^{+/+}$ and *Perk*$^{-/-}$ MEFs treated for 6 h with thapsigargin (Tg; 500 nM), BtdCPU (10 μM), and/or halofuginone (HF; 100 nM). There are 6 individual tabs in this workbook describing *h*$^{+/+}$ MEF treated with Tg, *Perk*$^{+/+}$ MEFs treated with BtdCPU, *Perk*$^{+/+}$ MEFs treated with HF, *Perk*$^{-/-}$ MEFs treated with Tg, *Perk*$^{-/}$MEFs treated with BtdCPU, and *Perk*$^{-/}$ MEFs treated with HF.

Table S4. (Supplement to Figure 2 and Figure S2. Pharmacologic ISR activators rescue ISR signaling in *Perk*-deficient MEFs). Expression, measured by RNAseq, of genes regulated by stress-responsive signaling pathways including the unfolded protein response (UPR), integrated stress response (ISR), heat shock response (HSR), oxidative stress response (OSR), and NFκB inflammatory response in *Perk*$^{+/+}$ and *Perk*$^{-/-}$ MEFs treated for 6 h with thapsigargin (Tg; 500 nM), BtdCPU (10 μM), and/or halofuginone (HF; 100 nM).
